# Study on the multi-parameter combination analysis and quantitative evaluation of the efficacy of extracorporeal shock wave therapy for plantar fasciitis

**DOI:** 10.3389/fbioe.2025.1681337

**Published:** 2025-12-18

**Authors:** Jing Zhao, Yongfei Du, Zhijie Xiang, YuZhe Tan, Jiayue Hu, Liu Yang, Haicheng Wei

**Affiliations:** 1 School of Information Engineering, NingXia University, Yinchuan, China; 2 School of Electrical and Information, North Minzu University, Yinchuan, China; 3 School of Medical Technology, North Minzu University, Yinchuan, China

**Keywords:** extracorporeal shock wave therapy, plantar fasciitis, principal component analysis, gait analysis, efficacy evaluation

## Abstract

**Background:**

Extracorporeal shock wave therapy (ESWT) is an effective non-invasive treatment for plantar fasciitis (PF) that relieves chronic pain, promotes tissue healing, and improves function, and is particularly suitable for cases unresponsive to conservative management. Compared with surgery, ESWT is less invasive, more cost-effective, and has demonstrated favorable long-term outcomes.

**Aim of the study:**

To address the limited reliability of subjective scales and the clinical translation difficulties of laboratory gait analysis in current efficacy evaluation of ESWT for PF, we propose a monocular-vision, multi-parameter combination framework for multidimensional decoupling assessment of gait dynamics.

**Methods:**

Using a monocular RGB camera, we recorded a total of 633 gait videos from 23 PF patients before and after ESWT intervention. Lower-limb kinematic parameters were extracted with the CtransPose pose-estimation algorithm and dimensionality reduction and reconstruction were performed by principal component analysis (PCA). Five core functional dimensions explaining a cumulative variance of 89.18% were successfully extracted, and a theoretical framework for multidimensional coupling of gait features was established.

**Results:**

The primary efficacy dimension (PC1) improved significantly after treatment: step length increased from 0.33 [0.30, 0.39] m to 0.37 [0.29, 0.44] m, stride length increased from 0.59 [0.52, 0.69] m to 0.63 [0.55, 0.75] m, and walking speed increased from 0.59 [0.48, 0.67] m/s to 0.62 [0.55, 0.75] m/s (all P < 0.05). The coordinated improvement of these objective parameters suggests that ESWT may effectively restore basic propulsion-phase locomotor function by alleviating pain. The gait-optimization dimension (PC3) reflected a pattern of improved gait efficiency, characterized by significantly increased walking speed (P < 0.001) while cadence remained unchanged, reflecting release of pain-avoidance strategies. The swing-control dimension (PC5) showed a significant reduction in maximum swing angle (P = 0.003), indicating a favorable transformation in swing control mechanisms.

**Conclusion:**

By establishing an objective assessment method centered on PC1 with PC3 and PC5 as sensitive indicators, this study provides a new paradigm for quantitative ESWT efficacy evaluation and individualized rehabilitation planning.

## 1 Introduction

Plantar fasciitis (PF) is a common and debilitating musculoskeletal disorder, affecting approximately 10% of the general population at some stage in their lifetime (Alhakami et al., 2024). The primary clinical symptoms manifest as heel pain upon weight-bearing after morning awakening or prolonged rest, with patients typically presenting a pain-avoidance gait (Richer et al., 2022). Studies have shown that foot pain and dysfunction (assessed via Foot Function Index (FFI)) in PF patients negatively impact their balance ability and gait, increasing fall risk (Dudoniene et al., 2023). Therefore, early and effective treatment for PF patients is crucial to alleviate foot pain, improve balance and gait stability, and reduce fall risk.

Due to its high safety and patient tolerance, ESWT is widely used in clinical interventions for chronic tendinopathies and plantar fasciitis (Poenaru et al., 2022; Moghtaderi et al., 2014). Based on physical characteristics and modes of action, shock wave therapy is generally categorized into focused (focused ESWT, ESWT) and radial (radial ESWT, R-ESWT) modalities (Gomulec, 2011; Król et al., 2009). ESWT is characterized by deeper penetration, short pulse rise time, steep peaks, and high pulse energy, with frequencies typically in the 1–22 Hz range and peak pressures that can be very large in the focal zone (Rompe et al., 2005; Speed et al., 2002); radial ESWT, in contrast, delivers lower energy, lower impact forces, and shallower penetration (Thiel, 2001; Gerdesmeyer and Weil, 2007). The two modalities differ clinically as well: ESWT typically uses higher energy flux densities (reported ranges ≈0.09–0.55 mJ/mm^2^) with less frequent sessions (e.g., once weekly), whereas R-ESWT tends to employ lower energy densities (≈0.03–0.25 mJ/mm^2^) with higher session frequency (e.g., two to three times per week) (International Society for Medical Shockwave Treatment, 2024; Cennamo et al., 2020; Reilly et al., 2022).

High-energy shock waves rapidly transmit energy into tissues, which may stimulate cellular repair and regenerative processes. Adverse events are uncommon and generally transient, most frequently consisting of short-term procedural pain, localized swelling, ecchymosis, erythema, numbness, or paresthesia; serious complications such as nerve injury are rare (Cheng and Wang, 2015), (De la Corte-Rodríguez et al., 2023). Multiple clinical studies have demonstrated that ESWT effectively reduces pain and functional impairment in PF patients (Tenforde et al., 2022), (Schroeder et al., 2021). As a promising non-invasive therapy, ESWT has shown durable benefits in certain cohorts—for example, in amateur runners where the Visual Analogue Scale (VAS) pain scores decreased substantially and low recurrence rates were observed at long-term follow-up—supporting sustained therapeutic effects compared with some alternative treatments (Kapusta and Domżalski, 2022). However, current evaluation of ESWT efficacy primarily relies on subjective pain scales (e.g., VAS) and patient-reported outcome measures. For instance, a randomized controlled trial by Loubiri et al. (2025) assessing radial rESWT for PF reported significant reductions in VAS scores at the end of treatment, 1 month, 3 months, and 6 months in both groups (p < 0.001). Similarly, a retrospective observational study by Rayo-Pérez et al. (2025) comparing conservative treatments for plantar fasciitis in 113 patients found that the ESWT group exhibited a decrease in VAS scores from a baseline of 7.7 to 1.8 at 6 months (p < 0.001). In contrast, a prospective randomized controlled trial by Ong et al. (2025) comparing ESWT combined with physical therapy versus physical therapy alone in 36 patients with acute plantar fasciitis showed no significant difference in VAS scores between the two groups at 3 months (ESWT group: 5.5 at baseline to 4.7 at 3 months). Although such measures can provide information on pain intensity, they fail to objectively quantify neuromuscular recovery or capture the spatiotemporal evolution of gait dynamics (Afzal et al., 2024). Such single-dimensional tools are susceptible to patient cognition, mood, and observer bias, limiting their reproducibility and objectivity (Grčić et al., 2013). Gait dynamics analysis provides an important objective quantification approach: prior gait studies have revealed statistically significant alterations in multiple gait-dynamic parameters in PF patients, and even outside acute flare-ups patients frequently exhibit an adaptive “antalgic gait,” such as reduced walking speed to offload plantar pressure (Cervera-Garvi et al., 2023).

The aim of the present study is to address limitations of subjective outcome measures and the clinical translation barrier of laboratory gait assessment by proposing a monocular-vision-based, multi-parameter combinatorial framework for gait-dynamics-based, multidimensional evaluation of ESWT efficacy in patients with plantar fasciitis.

## 2 Experimental process and methods

**FIGURE 1:**
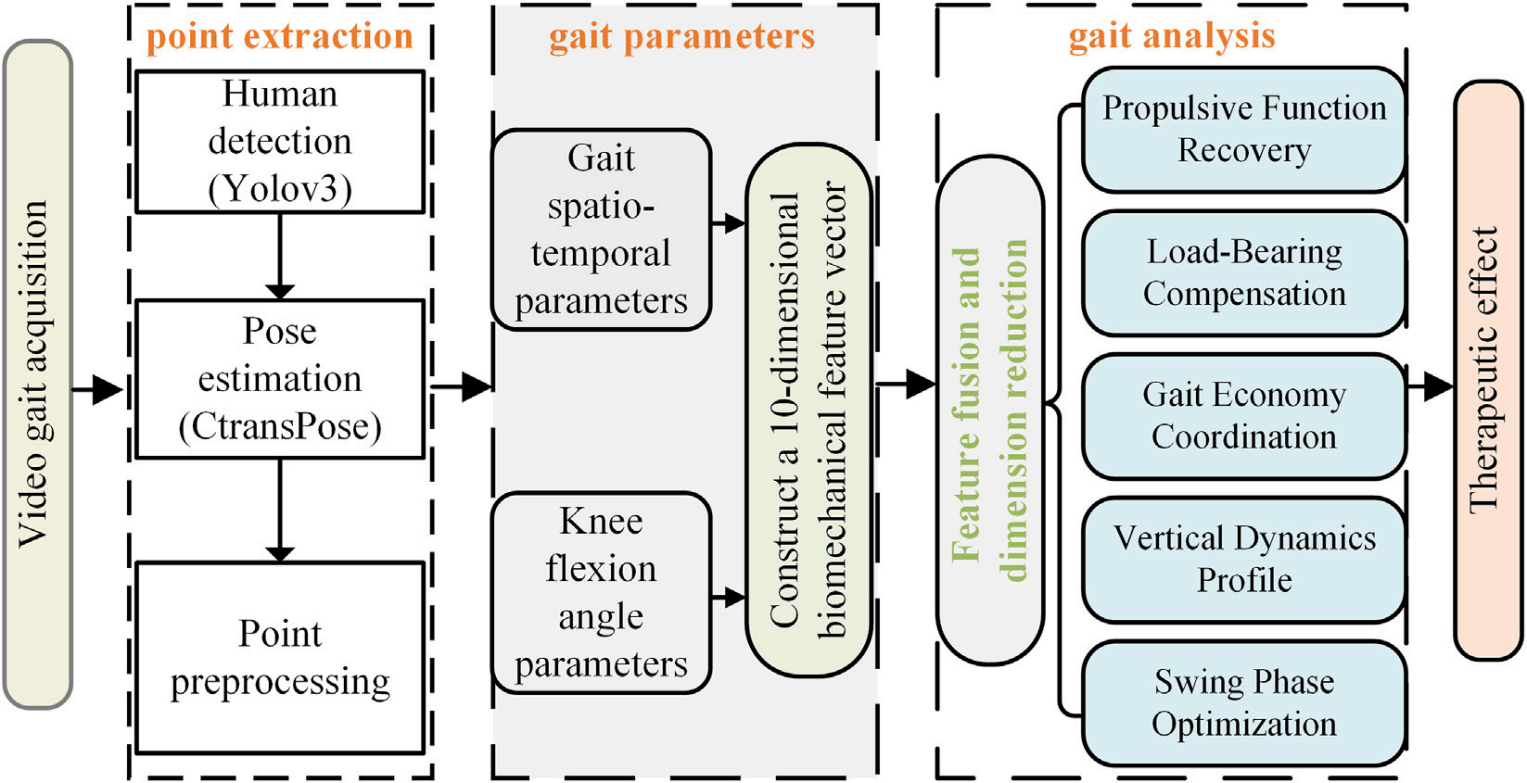
Framework of the gait parameter quantitative assessment system.

The overall system block diagram of the proposed method is shown in Figure 1. The implementation process of this method can be divided into five stages: first, natural gait sequence video data of PF patients is captured via high-frame-rate camera equipment; then, based on the YOLOv3 model, human body detection and localization in the video stream are achieved, and the human pose estimation algorithm CtransPose is applied within the detection bounding box regions to accurately extract two-dimensional coordinate data of lower-limb kinematic chain bone joint points in consecutive video frames; through a method combining kinematic interpolation and data normalization, the original joint point coordinates undergo temporal smoothing processing and spatial standardization preprocessing to eliminate acquisition noise and establish a unified motion analysis benchmark; building on this, a gait spatiotemporal parameter calculation model based on the motion trajectory of the heel joint point is constructed to achieve precise quantification of basic kinematic parameters (step length, step cadence, step speed), and a knee joint kinematics analysis module is further integrated to extract 10 key biomechanical parameters including the peak knee flexion angle and the time proportion of stance phase/swing phase in the gait cycle; data dimensionality reduction is performed via Principal Component Analysis to screen out the feature parameter set with the most significant ESWT therapeutic intervention effect (cumulative variance contribution rate >80%); finally, based on the statistical differences in principal component scores before and after PF treatment, the biomechanical improvement effects of ESWT on the patient’s gait pattern are systematically evaluated.

### 2.1 Gait quantitative assessment parameters

Gait quantitative assessment parameters are key indicators in gait analysis, used to describe and evaluate an individual’s walking pattern (Khan et al., 2024). This study selected spatiotemporal gait parameters (step length, gait speed, gait cadence) and knee flexion angle to objectively assess functional impairment and treatment efficacy in PF patients. Through these spatiotemporal metrics, the efficiency and coordination of lower limb movement throughout the entire walking cycle can be characterized.

The flexion angle of the knee joint is an important indicator for evaluating the mobility and health status of the knee joint. The variation curve of the knee joint angle under a complete gait cycle is generally bimodal (Wei et al., 2023). Take the same side foot as an example. Suppose in a frame of pictures, the coordinates of the hip joint are (x1,y1), the coordinates of the knee joint are (x2, y2), and the coordinates of the ankle joint are (x3,y3). The calculation process of the knee flexion angle is as follows:

First, calculate the Euclidean distance between each pair of the hip joint, knee joint, and ankle joint, that is:

$$d_{13}=\sqrt{{\left(x_{1}-x_{3}\right)}^{2}+{\left(y_{1}-y_{3}\right)}^{2}}$$ (1)

$$d_{12}=\sqrt{{\left(x_{1}-x_{2}\right)}^{2}+{\left(y_{1}-y_{2}\right)}^{2}}$$ (2)

$$d_{23}=\sqrt{{\left(x_{2}-x_{3}\right)}^{2}+{\left(y_{2}-y_{3}\right)}^{2}}$$ (3)

Then the angle of the knee joint is obtained by the cosine theorem, that is:

$$\theta =180{}^{\circ}-\cos^{-1}\left(\frac{d_{12}^{2}+d_{23}^{2}-d_{13}^{2}}{2d_{12}d_{23}}\right)$$ (4)

Perform the same processing on each frame of the video to obtain a continuous sequence of knee joint angle change signals. The gait characteristics are obtained through the analysis of the signal feature points.

### 2.2 Principal component analysis

PCA is an unsupervised linear dimensionality reduction and feature extraction technique (Kurita, 2021). Its core objective is to transform correlated original variables into fewer uncorrelated principal components via orthogonal transformation. These components are ordered by descending variance: the first retains maximum data variance, while subsequent components sequentially maximize remaining variance under orthogonality constraints, achieving data compression and feature extraction. The algorithm details are as follows:

To eliminate scale differences, original variables undergo Z-score standardization to achieve a distribution with mean 0 and variance 1. PCA projects data onto a new coordinate system via linear transformation. The basis vectors of this system are eigenvectors of the original data’s covariance matrix. Let Z be the standardized data matrix. The covariance matrix C is computed as Equation 5, and its eigendecomposition is given by Equation 6:

$$C=\frac{1}{n-1}Z^{T}Z$$ (5)

$$C=V\Lambda V^{T}$$ (6)

where C is the covariance matrix of standardized data, Λ is a diagonal matrix composed of eigenvalues $\lambda_{1},\lambda_{2},...,\lambda_{p}$ , and V is the eigenvector matrix. The principal components are calculated as follows:

$${\text{PC}}_{k}=Zv_{k}$$ (7)

Here, $v_{k}$ is the eigenvector corresponding to the k-th largest eigenvalue. The contribution rate and cumulative contribution rate of the principal components are calculated. Then, based on the cumulative contribution rate, k principal components are extracted. $\lambda_{k}$ represents the variance of the k-th principal component (PCk), p denotes the total number of original variables. We then calculate the variance contribution rate and the cumulative contribution rate of each principal component, and select the first k principal components under the requirement that the cumulative contribution rate exceeds 85%:

$$\eta_{k}=\frac{\lambda_{k}}{\sum_{i=1}^{p}\lambda_{i}}$$ (8)

$$\Gamma_{k}=\frac{\sum_{i=1}^{k}\lambda_{i}}{\sum_{i=1}^{p}\lambda_{i}}\times 100\%$$ (9)

## 3 Experimental design

### 3.1 Data description

The present study included 23 patients with PF who presented to the Ningxia Hui Autonomous Region People’s Hospital and met the study inclusion criteria. Patients were aged 42–72 years (mean ± SD: 52 ± 11 years). Inclusion criteria were: 1. disease duration >3 months consistent with the clinical diagnosis of chronic plantar fasciitis (Koc et al., 2023); 2. a Visual Analog Scale (VAS, 0–10) pain score >4 for the first step in the morning; 3. clinically evident symptom improvement following ESWT intervention; 4. no prior foot or ankle surgery or history of significant trauma; 5. strict restriction of medication use that could confound treatment effects—specifically, initiation of new non-steroidal anti-inflammatory drugs (NSAIDs) or systemic/local corticosteroids was avoided for at least 2 weeks surrounding the treatment period; 6. no prior physical therapy prior to enrollment. The sample was a convenience sample of consecutive patients who met these criteria. The relatively small sample size is acknowledged as a limitation and is discussed in the Limitations section. Exclusion criteria included pregnancy, presence of peripheral neuropathy, peripheral vascular disease, coagulopathy, or local infection. The study protocol was approved by the Ethics Committee of the People’s Hospital of Ningxia Hui Autonomous Region (Approval No.: 2024-KJCG-001). Written informed consent was obtained from all participants before enrollment.

Intervention consisted of ESWT. Patients received 1 to 5 weekly treatment sessions (one session per week). Treatment parameters were set as follows: application frequency approximately 1000 pulses/min and an energy flux density of 0.25 mJ/mm^2^. No local anesthesia was used during the procedures. These parameters were selected with reference to previously published studies and relevant literature (Ali et al., 2014). Adverse events were recorded at each visit and managed according to standard clinical procedures.

Baseline demographic and clinical data extracted from medical records included age, sex, affected side, height, weight, comorbidities. Pain was assessed using the Visual Analog Scale (VAS) at baseline and follow-up visits; medication use and adherence to any prescribed rehabilitation exercises were documented at each assessment.

Gait data after each ESWT session were collected strictly within a T + 30-minute time window (i.e., within ±5 min of 30 min after treatment completion). The post-treatment procedure was: patients were escorted to a standardized recovery area and seated at rest for 25 min—a period chosen to allow resolution of transient pain-related metabolic peaks and to reduce short-lived muscle tension associated with the procedure (Jeon et al., 2019; Sampson et al., 2023) — after which gait testing was performed under researcher supervision.

All ESWT procedures were performed by a rehabilitation physician with more than 5 years of clinical experience, who holds a national medical license and is skilled in the operation of ESWT equipment and treatment protocols.

### 3.2 Experimental procedure

**FIGURE 2:**
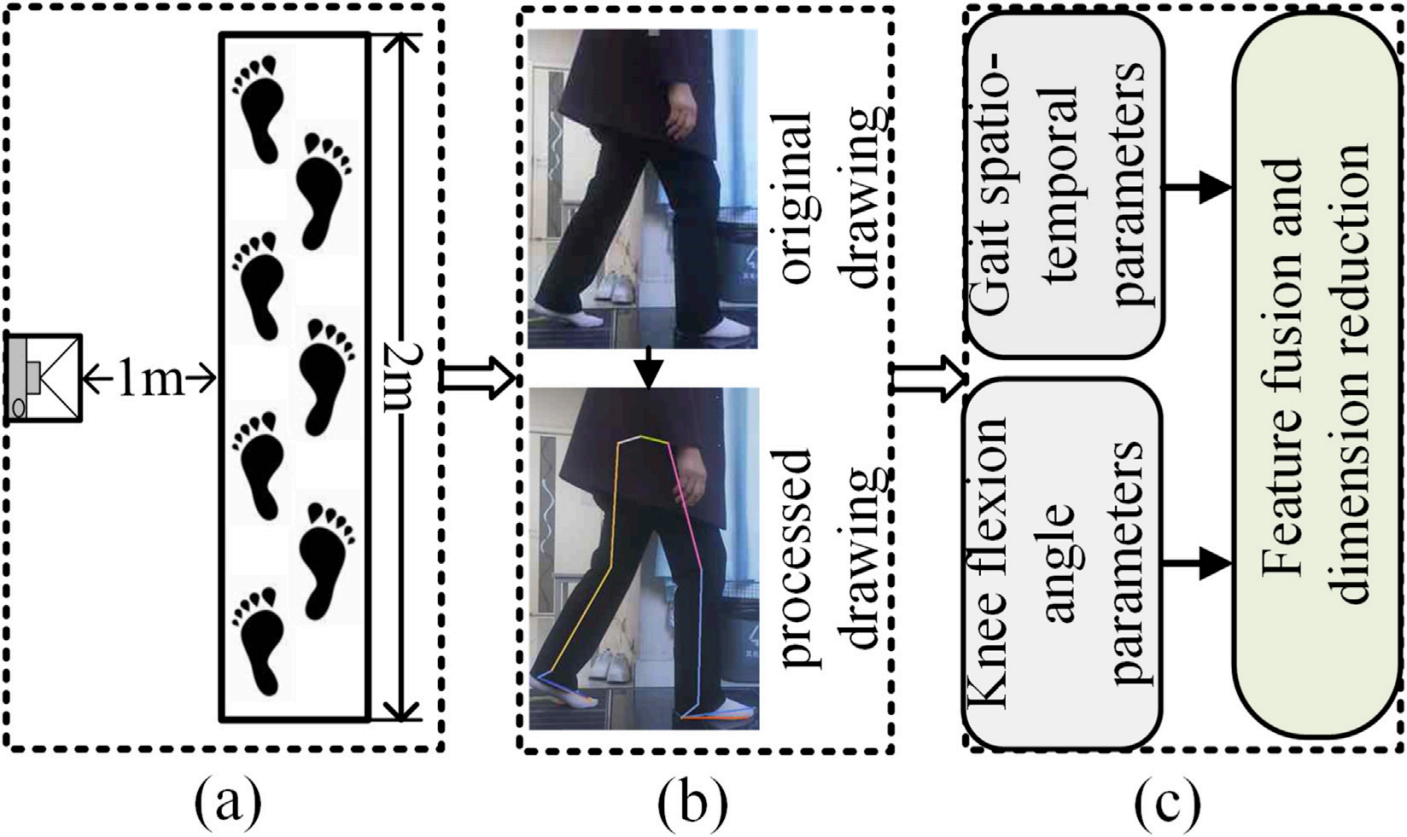
Experimental process diagram. **(a)** Shooting scene, **(b)** Key point recognition, **(c)** Parameter analysis.

This experiment was conducted indoors, and the walking videos of the subjects were captured by a monocular RGB camera placed on a tripod. The camera resolution is 1920 × 1080, the frame rate is 30fps, and the focal length is 1 mm. To track the movement process of the subjects, in the formal test, the subjects walked barefoot on a 2-meter by 0.5-meter straight path with a natural gait, eliminating the interference of arch support such as insoles and high heels. The path was set on a flat ground and paved with standard anti-slip rubber pads. The environmental conditions were good, avoiding the interference of strong light and reflection. During each test, the patient walked along a straight line at a natural and comfortable pace, completing three round trips from two opposing directions (left-to-right and right-to-left) to ensure sufficient data collection and to average out accidental errors. Each one-way walk was recorded as a separate valid gait video. Accordingly, this study collected a total of 633 valid gait videos. As shown in Figure 2, it is the experimental process diagram.

### 3.3 Data processing

Based on the video-captured data, this study employed the CtransPose human pose estimation algorithm to extract skeletal joint coordinates frame-by-frame for PF patients before and after ESWT treatment. The data processing flow is as follows: 1. Data Preprocessing: Eliminate invalid data points and perform noise filtering; 2. Temporal Smoothing: Apply a moving average method to reduce random fluctuations; 3. Gait Event Identification: Define the gait cycle through heel strike and toe-off events; 4. Parameter Calculation: Parse spatiotemporal parameters such as step length and step speed based on the gait cycle (see Table 1 for detailed parameter definitions); 5. Knee Joint Kinematics Analysis: Utilize a median filter combined with a Savitzky-Golay filter to smooth and denoise angle data (Crenn et al., 2021); 6. Locate Local Extrema Points to Mark Key Angle Phases, such as the minimum flexion angle in the stance phase, the maximum flexion angle in the swing phase, and the proportion of swing phase duration. The definitions of the collected gait parameters are shown in Table 1:

**TABLE 1:** The definition of gait parameters.

Parameter	Definition (all during walking)
Stride	Longitudinal linear distance between two consecutive heel strikes of the same foot
Step_length	Longitudinal linear distance between the heel strike point of one foot and the heel strike point of the opposite foot
Step_height	Maximum vertical distance of the foot from the ground during the swing phase
Cadence	Number of steps per unit time (steps/min)
Speed	Step length divided by the step time
Maximum flexion angle in loading	The maximum flexion angle reached by the knee joint during the supporting phase
Minimum flexion angle in stance	The minimum flexion angle of the knee joint during the supporting phase
Maximum flexion angle in swing	The maximum flexion angle reached by the knee joint during the swing phase (Perry and Burnfield, 2024), (Darter et al., 2019)
Stance phase percentage	Duration from ground contact to toe-off of one lower limb as a percentage of the total gait cycle
Swing phase percentage	Duration from toe-off to next ground contact of one lower limb as a percentage of the total gait cycle

During this process, 4489 continuous gait cycles were initially extracted, and 300 invalid cycles (including occlusion, missing key points, or incomplete gait data) were excluded, resulting in 4189 gait cycles ultimately included in the analysis, with a valid data proportion of 93.31%. To conduct longitudinal paired analysis, the data were matched at the individual level. A total of 225 high-quality baseline-intervention paired datasets were successfully constructed, with a pairing success rate of 84.9%. For the pre- and post-therapy paired analysis, videos recorded before and after each therapy session were paired. The study initially screened 265 baseline-intervention paired datasets, excluding 40 invalid pairs due to loss to follow-up or incomplete data (an exclusion rate of 15.1%). Ultimately, 225 longitudinal comparisons were completed, with a retention rate of 84.9% for the paired data.

### 3.4 Statistical analysis

To analyze the within-group differences in gait parameters of plantar fasciitis patients before and after extracorporeal shockwave therapy, this study first conducted the Shapiro–Wilk normality test on the paired differences of each parameter. As the variables did not follow a normal distribution, the Wilcoxon signed-rank test was used for within-group comparisons. Data are presented as “median (Q1–Q3).” The significance level was set at α = 0.05, and symbols were used to denote significance: *P < 0.05, **P < 0.01, ***P < 0.001. The p-values for multiple comparisons were adjusted using the Benjamini–Hochberg FDR correction to control the false discovery rate. Effect sizes (r) were interpreted according to the following criteria: small effect ≥0.1, medium effect ≥0.3, and large effect ≥0.5. All statistical analyses were performed using the statsmodels and scipy libraries in a Python 3.8.3 environment.

## 4 Result analysis

The intra-group differences in gait parameters of patients with PF before and after ESWT treatment are shown in Table 2.

**TABLE 2:** Comparison of gait parameters before and after treatment.

Parameter	Pre-treatment	Post-treatment	p-value	FDR-corrected p-value	Effect size r
Stride(m)	0.59 [0.52, 0.69]	0.63 [0.55, 0.75]	0.002**	0.004	0.208
Step_length(m)	0.33 [0.30, 0.39]	0.37 [0.29, 0.44]	<0.001***	<0.001	0.260
Step_height(m)	0.02 [0.01, 0.03]	0.02 [0.01, 0.03]	0.309	0.387	0.068
Cadence(steps/min)	106.57 [98.32, 113.41]	106.57 [93.98, 114.22]	0.381	0.424	0.058
Speed(m/s)	0.59 [0.48, 0.67]	0.62 [0.55, 0.75]	<0.001***	<0.001	0.347
Max_weight_bearing_angle(°)	17.19 [12.83, 19.95]	15.23 [11.67, 22.19]	0.253	0.361	0.076
Min_stance_phase_angle(°)	7.75 [5.50, 9.40]	7.19 [4.24, 10.71]	0.867	0.867	0.011
Max_swing_phase_angle(°)	50.51 [45.23, 55.43]	49.36 [41.09, 56.14]	0.003**	0.005	0.199
Percentage_of_stance_phase(%)	40.73 [35.63, 44.05]	44.12 [37.92, 52.08]	<0.001***	<0.001	0.306
Percentage_of_swinging_phase(%)	59.27 [55.95, 64.37]	55.88 [47.92, 62.08]	<0.001***	<0.001	0.306

Spatiotemporal parameter analysis showed significant changes in gait cycle parameters after ESWT treatment: stride length (0.59 [0.52, 0.69] m to 0.63 [0.55, 0.75] m (p = 0.002; r = 0.208)), step length (0.33 [0.30, 0.39] m to 0.37 [0.29, 0.44] m (p < 0.001; r = 0.260)), and walking speed (0.59 [0.48, 0.67] m/s to 0.62 [0.55, 0.75] m/s (p < 0.001; r = 0.347)) all increased with small to moderate effect sizes. These changes are consistent with a transition towards a more efficient gait pattern, characterized by increased stride length, step length, and speed. Meanwhile, cadence showed no significant change (106.57 [98.32, 113.41] to 106.57 [93.98, 114.22] steps/min; p = 0.381).

Regarding kinematic parameters, the proportion of the stance phase significantly increased from 40.73% [35.63, 44.05] to 44.12% [37.92, 52.08] (p < 0.001; r = 0.306). Correspondingly, the proportion of the swing phase decreased from 59.27% [55.95, 64.37] to 55.88% [47.92, 62.08] (p < 0.001; r = −0.306). In the swing phase, the maximum swing phase angle significantly decreased from 50.51° [45.23, 55.43] to 49.36° [41.09, 56.14] (p = 0.003; r = 0.199). Other kinematic angles, including the minimum stance phase angle (p = 0.867) and maximum loading angle (p = 0.253), showed no statistically significant differences with effect sizes approaching zero.

**FIGURE 3:**
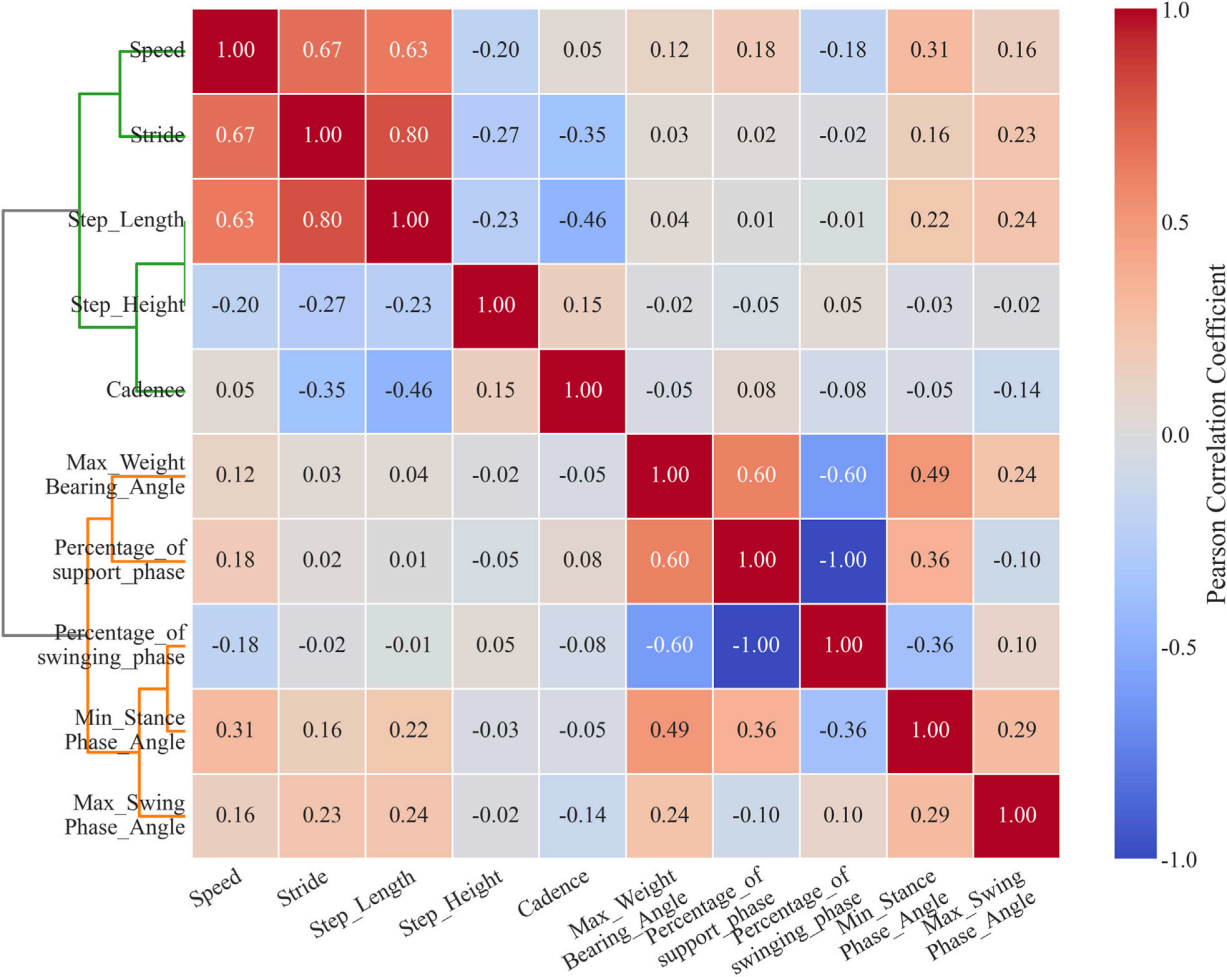
Gait parameter correlation matrix based on hierarchical clustering.

To perform automated grouping and correlation analysis of gait parameters, thereby objectively revealing post-treatment improvements and potential residual abnormal patterns, this study constructed a correlation matrix of gait parameters through hierarchical clustering using Ward’s linkage. The dendrogram on the left side of Figure 3 illustrates the clustering relationships among parameters, while the heatmap in the middle displays the correlation coefficients. The results show that gait parameters can be divided into two main clusters; the green cluster contains all spatiotemporal parameters: walking speed, stride length, step length, step height, and cadence. Within this cluster, speed was moderately positively correlated with stride length (r = 0.67) and step length (r = 0.63). Meanwhile, stride length was weakly negatively correlated with step height (r = −0.27) and cadence (r = −0.35).

The orange cluster includes all kinematic parameters: maximum flexion angle during loading response, minimum flexion angle during stance phase, maximum flexion angle during swing phase, proportion of stance phase, and proportion of swing phase. Within this cluster, the maximum flexion angle during loading response was moderately positively correlated with the proportion of stance phase (r = 0.6). The minimum flexion angle during stance phase was weakly negatively correlated with the proportion of swing phase (r = −0.36). The proportion of stance phase and proportion of swing phase were perfectly negatively correlated (r = −1.00).

To effectively integrate information and eliminate parameter redundancy, this study employed PCA as a linear dimensionality reduction method: First, the level of multicollinearity was evaluated by calculating the Variance Inflation Factor (VIF) and condition index. The results indicate that the stance phase percentage and swing phase percentage exhibit perfect collinearity (r = −1.00), representing a mathematically complementary relationship (their sum is constant at 100%). We chose to exclude both variables rather than retaining either one for the following reason: retaining either variable would introduce a constant-sum constraint into the dataset, artificially inflating the explained variance of the first principal component while complicating the biological interpretation of subsequent principal components, without adding any independent information (Filzmoser et al., 2009), it remains essentially a redundant parameter that does not contribute additional explanatory power to the model. therefore, they were removed from subsequent analyses. The average variance inflation factor (VIF = 2.22 < 5) and maximum condition index (3.98 < 10) of the remaining gait parameters indicated that conventional multicollinearity issues were not significant. Eigenvalue analysis showed that the first five principal components explained 89.18% of the variance as shown in Table 3, supporting the use of PCA for dimensionality reduction. The remaining 8 parameters after removing the perfectly collinear parameters underwent Z-score standardization to meet a distribution with mean = 0 and variance = 1, thereby eliminating scale effects. Second, the factor loading matrix was optimized through orthogonal rotation using the Varimax method to enhance the interpretability of the factor structure. Then, for each principal component, parameters with absolute factor loadings ≥0.5 were screened as significant contributing factors; finally, based on the common characteristics of parameters with high loadings in the principal components and combined with the research context, the principal components were named and interpreted. The high-loading variables (|loadings|≥0.5) for principal components PC1-PC5 are shown in Table 4, with significant loading parameters marked by asterisks (*).

**TABLE 3:** Eigenvalue analysis.

Principal	Characteristic value	Conditional index	Variance contribution Rate(%)	Cumulative variance contribution rate (%)
PC1	2.892	1.00	36.15	36.15
PC2	1.528	1.38	19.11	55.26
PC3	1.065	1.65	13.31	68.57
PC4	0.906	1.79	11.32	79.89
PC5	0.743	1.97	9.29	89.18
PC6	0.479	2.46	5.99	95.17
PC7	0.204	3.77	2.55	97.72
PC8	0.183	3.98	2.29	100.00

**TABLE 4:** Principal component load matrix.

Parameter	PC1	PC2	PC3	PC4	PC5
Stride	0.51*	−0.23	−0.08	0.13	−0.04
Step_Length	0.52*	−0.21	0.04	0.16	−0.12
Step_Height	−0.21	0.20	0.01	0.89*	−0.35
Cadence	−0.26	0.20	−0.78*	0.01	0.28
Speed	0.45	−0.03	−0.54*	0.09	−0.04
Max_Weight_Bearing_Angle	0.16	0.62*	0.11	−0.27	−0.27
Min_Stance_Phase_Angle	0.27	0.57*	−0.06	−0.09	−0.23
Max_Swing_Phase_Angle	0.25	0.34	0.27	0.29	0.81*

PC1, as the core dimension representing mechanical propulsion function, demonstrated significant absolute loadings on step length (|loading| = 0.52), stride length (|0.51|), and speed (|0.45|). These parameters showed significant improvement post-treatment (p < 0.01), coupled with a 6.98% prolongation of the stance phase (p < 0.001).

PC2 primarily reflects knee flexion pattern during weight acceptance. This component showed high absolute loadings on maximum weight-bearing angle (|0.62|) and minimum stance phase angle (|0.57|), indicating co-variation of knee flexion angles during the loading response and mid-stance phases. Pre-treatment values for maximum weight-bearing angle (17.19° [12.83, 19.95]) and post-treatment values (15.23° [11.67, 22.19]) both fell within established normative ranges for healthy adults (Perry and Burnfield, 2010).

PC3 unveils a gait optimization dimension: This component showed substantial loadings on cadence (|0.78|) and speed (|0.54|). Empirical data showed that cadence did not change significantly after treatment (−0.81%, p = 0.381), while speed increased significantly (+6.67%, p < 0.001).

PC4 characterizes a vertical dynamics dimension: This component was predominantly defined by step height (|0.89|), which showed no significant difference before and after treatment (0.02 [0.01, 0.03] changed to 0.02 [0.01, 0.03] m, p = 0.309).

PC5 independently represents a swing control dimension: This component was primarily governed by the maximum swing angle (|0.81|). This variable decreased significantly by 2.3% post-treatment (from 50.51° to 49.36°, p = 0.003).

## 5 Discussion

This study proposes a monocular-vision and multi-parameter combinatorial analysis–based framework for multidimensional decoupled evaluation of gait dynamics, designed to objectively assess the rehabilitative effects of ESWT in patients with plantar fasciitis. Specifically, the core dimension represented by PC1 (step length and walking speed) increased significantly after treatment (p < 0.001). This pattern aligns with the hypothesis that the plantar fascia restores its elastic energy storage-release function during horizontal propulsion (Wager and Challis, 2016). Correlation analysis further revealed that improvements in speed were driven by spatial parameters rather than cadence. This suggests that shockwave therapy likely reduced plantar pain, allowing patients to adopt a longer stride length. In the gait-optimization dimension (PC3), a trend toward increased speed without a significant change in cadence (p = 0.381) was observed. This represents a trade-off adjustment between rhythm and efficiency: patients achieved better overall propulsion post-operation without accelerating their rhythm. Swing control improved: a significant decrease in the maximum swing angle was observed in the swing-control dimension (PC5) (p = 0.003), suggesting improved propulsive-phase efficiency. The maximum weight-bearing angle—the primary loading variable of PC2—remained stable from pre- to post-assessment (17.19° vs. 15.23°, p = 0.253), falling within the typical 15°–20° range reported for healthy gait (Chao et al., 1983). Similarly, vertical dynamic performance, represented by PC4 (step height), showed no significant change (p = 0.309). Collectively, the PCA model accounted for 89.18% of the total gait variance.

ESWT significantly improved the core functional dimensions of gait in patients with PF, a conclusion that aligns closely with existing literature: the synergistic enhancement in stride length, step length, and walking speed post-treatment (p < 0.001) indicates normalization of foot propulsion function following pain relief. This is consistent with studies employing specialized equipment; for instance, the pilot study by Kamonseki et al. (2020) reported immediate improvements in walking biomechanics among patients with plantar heel pain after ESWT, including an increase in walking speed attributed to reduced inflammation in the plantar fascia and enhanced propulsion phase efficiency. Similarly, the randomized controlled trial by Saleh et al. (2024) demonstrated a 15%–20% increase in walking speed in the ESWT group post-treatment (p < 0.01); existing research has preliminarily confirmed the ameliorative effects of ESWT on gait parameters in PF patients (Jiang et al., 2021). Beyond spatiotemporal changes, the kinematic analysis revealed deeper functional adjustments. The significant increase in the stance phase proportion indicates prolonged single-leg support time, which may enhance dynamic balance control and provide a more stable platform for center of gravity adjustment. Notably, our correlation matrix revealed that the maximum flexion angle during loading response was moderately positively correlated with the stance phase proportion (r = 0.6). This reflects that patients might compensate for insufficient shock absorption in the ankle by increasing knee joint cushioning. The present study, utilizing a simplified system based on a monocular RGB camera, captured analogous patterns, thereby further validating its clinical applicability and circumventing the complexities associated with laboratory equipment.

In the gait optimization dimension (PC3), cadence showed no significant change (p = 0.381) despite increased speed, implying a “maintained cadence with improved gait efficiency” mode. Although this trend did not reach statistical significance, these comparisons indicate that ESWT’s role in strategic dimensions is more auxiliary, serving as a core component in comprehensive rehabilitation programs. In the strategic dimensions of gait, ESWT exhibited a positive adjunctive role, albeit with varying intensity across dimensions. In the swing control dimension (PC5), the maximum swing angle significantly decreased (p = 0.003). Considering other joint coordination parameters, this reduction can be interpreted as a potential indicator of improved hip-knee synergistic control, although it may also suggest that patients exhibit a slight tendency to reduce swing amplitude to maintain stability. Additionally, the weak negative correlation between stride length and step height observed in the cluster analysis suggests that some patients might retain a tendency towards a “shuffling gait” to avoid excessive tension in the plantar fascia during toe-off.

Despite ESWT’s efficacy in core and strategic dimensions, the present study also revealed limitations in knee flexion pattern during weight acceptance and vertical dynamic dimensions. PC2, which primarily captures knee flexion during weight acceptance, showed no significant change in maximum weight-bearing angle (pre: 17.19°; post: 15.23°; p = 0.253). These values remained within normal physiological limits throughout (15°–20°), indicating that this aspect of knee function was not pathological either before or after treatment. Similarly, PC4 (vertical dynamics) exhibited no significant change in step height (p = 0.309). Although toe clearance in healthy gait is typically 1–2 cm, the observed minor deviation in this cohort may reflect a residual cautious strategy rather than a clear pathological adaptation, warranting further investigation in future studies. These limitations underscore that while ESWT can alleviate symptoms, rectification of chronic compensations necessitates personalized extensions (Yoo et al., 2017; Chang et al., 2014).

From a technical perspective, the present study confirmed the efficiency of monocular vision combined with PCA in extracting therapeutic efficacy dimensions. This method features a low equipment threshold and, compared to traditional approaches relying on specialized laboratory equipment (e.g., Vicon system (Chen et al., 2022), Zebris treadmill (Brachman et al., 2020), AMTI force plates (Van Criekinge et al., 2023), or pressure measurement platforms (Wang B. et al., 2024)), is more amenable to clinical dissemination. This approach is congruent with the study by Tsuchida et al. (2022), which employed PCA to compare gait features in frail versus non-frail elderly women, and resonates with the autoencoder method developed by Wang S. J. et al. (2024) for the Shriners Gait Index; likewise, Tabashum et al. (2022) explored the utility of PCA components versus autoencoder features in assessing prosthetic performance in individuals with lower-limb amputation. Collectively, these studies validate PCA’s reliability in distilling clinical indicators from multidimensional data, and the current framework extends its application in ESWT evaluation.

Study Strengths: The strength of this study lies in its innovative multidimensional assessment framework, which not only achieves lossless dimensionality reduction from high-dimensional gait data to low-dimensional clinical features but also pioneers the application of monocular vision technology for quantifying ESWT efficacy, avoiding reliance on expensive equipment. The method explained 89.18% of the gait variance, providing a reproducible tool for objective monitoring. Owing to these technical characteristics, the proposed assessment framework possesses strong generalizability, enabling efficient and precise evaluation and dynamic tracking of PF rehabilitation outcomes in primary clinical settings.

Limitations: This study has the following limitations: The limited sample size and follow-up period prevent capturing long-term gait trajectories and potentially overlook the effects of chronic recurrence. The focus on the biomechanical model without analyzing correlations between VAS/FFI scores and PCA indicators restricts the integration of subjective and objective assessments. The outcomes are based on patient self-reporting rather than objective examinations like ultrasound or blood flow measurements. Consequently, the obtained data are subjective and potentially inaccurate. Therefore, caution is warranted when interpreting our findings.

## 6 Conclusion

Building upon gait multi-parameter analysis, this study establishes a multidimensional quantitative assessment system for evaluating ESWT efficacy, providing objective biomechanical evidence for the rehabilitation assessment of plantar fasciitis. This system offers clinicians more comprehensive therapeutic feedback, facilitating the optimization of personalized treatment plans. Based on the above findings, we propose the following clinical recommendations: incorporate the current PCA-based framework into routine follow-up assessments, using PC1 as a sensitive and comprehensive indicator to monitor overall treatment response to ESWT; adopt low-cost monocular vision systems in primary care and rehabilitation settings to facilitate objective, accessible gait evaluation for plantar fasciitis patients. In the future, this method holds promise for standardizing PF rehabilitation pathways and improving patient outcomes.

## Data Availability

The raw data supporting the conclusions of this article will be made available by the authors, without undue reservation.
